# Seeing the forest for the trees through metabolic scaling

**DOI:** 10.1093/pnasnexus/pgac008

**Published:** 2022-03-10

**Authors:** Igor Volkov, Anna Tovo, Tommaso Anfodillo, Andrea Rinaldo, Amos Maritan, Jayanth R Banavar

**Affiliations:** Department of Physics, The George Washington University, 20052 Washington, DC, USA; Dipartimento di Fisica “G. Galilei”, Università di Padova, 35131 Padova, Italy; Dipartimento Territorio e Sistemi Agro-Forestali, Università di Padova, 35020 Agripolis, Legnaro (PD), Italy; Laboratory of Ecohydrology, École Polytechnique Fédérale de Lausanne, CH-1015 Lausanne, Switzerland; Dipartimento di Ingegneria Civile , Edile e Ambientale, Università di Padova, 35131 Padova, Italy; Dipartimento di Fisica “G. Galilei”, Università di Padova, 35131 Padova, Italy; Consorzio Nazionale Interuniversitario per le Scienze Fisiche della Materia, Istituto Nazionale di Fisica Nucleare, 35131 Padova, Italy; Department of Physics and Institute for Fundamental Science, University of Oregon, 97403 Eugene, OR, USA

**Keywords:** Climate science, Scaling, Forests, Ecology, Carbon sequestration

## Abstract

We demonstrate that when power scaling occurs for an individual tree and in a forest, there is great resulting simplicity notwithstanding the underlying complexity characterizing the system over many size scales. Our scaling framework unifies seemingly distinct trends in a forest and provides a simple yet promising approach to quantitatively understand a bewilderingly complex many-body system with imperfectly known interactions. We show that the effective dimension, *D*_*tree*_, of a tree is close to 3, whereas a mature forest has *D*_*forest*_ approaching 1. We discuss the energy equivalence rule and show that the metabolic rate–mass relationship is a power law with an exponent *D*/(*D* + 1) in both cases leading to a Kleiber’s exponent of 3/4 for a tree and 1/2 for a forest. Our work has implications for understanding carbon sequestration and for climate science.

Significance Statement Forests are terrestrial ecosystems with a high degree of structural and functional diversity: in the tropics, there often are hundreds of coexisting plant species with different habitats and thousands of consumers, each of them with inter-specific relationships with plant species. A forest is, thus a complex many body system spanning multiple scales with imperfectly known temporally and spatially varying interactions. A pressing goal in ecology is to understand how general trends and patterns emerge in spite of such complexity. It is of great societal relevance to understand whether the approximate scale-free behavior of a forest can result in underlying simplicity in a bewilderingly complex system that is directly relevant to climate science.

## Introduction

Forests cover about 30% of the land surface and have an essential role in regulating the Earth-climate system. They are responsible for about 50% of the total net primary productivity (NPP) and they act as a climate cooler because of evapotranspiration, while also affecting the planetary water cycle. They influence the energy budget of the planet by having a low surface albedo. All these functions are essentially determined by the amount of leaves within the forest community and by the length of the growing season. As a consequence, forest attributes (number of trees, tree size distributions, total leaf area, and so on) are key traits to be studied for understanding the effective role of tree communities in different sites and environmental conditions (1–3).

We build on the well-known empirical observations that trees span an enormous range of sizes from a seedling to a fully grown tree. Power law scaling (4, 5) relationships, also known as allometric scaling (6), are pervasive in tree communities. Here, we derive a unified theoretical framework for elucidating the role of geometry in determining the metabolic rate–mass relationship of a tree and the NPP–biomass density relationship of a forest.

When quantities in a system vary over many orders of magnitude, it is not uncommon that they exhibit power law scaling behavior with finite-size effects. We make the constructive hypothesis that such behavior holds and study the consequences, especially those that provide nontrivial links between seemingly disparate quantities. We find good accord with empirical data and prior expectations suggesting that our basic hypothesis may indeed be valid. Such scaling analysis is useful when the overarching behavior emerges despite the plethora of specific and often imprecisely known details.

We begin with a discussion of plant allometry and then turn to an understanding of the assembly of a forest. We will postulate power law relationships characterized by scaling exponents, an assumption that is supported by empirical data. As is well-known in the context of critical phenomena (4, 7, 8), we show that even here the exponents turn out to be related to each other linking seemingly distinct processes and phenomena. Unlike physical systems, here there is no fine-tuning involved and the power law behavior is only approximate but the trends are robust.

## Metabolic Scaling

The ability of a living organism to sustain itself, to grow, and to reproduce depends on its metabolic rate or energy available to it from its environment per unit time. An organism utilizes a fraction of the energy taken in and the rest is radiated out to the environment. Generally, the rate at which energy is radiated out by an organism is proportional to its surface area times the velocity with which energy is transported at its surface (9). Thus, the dependence of the metabolic rate of a living organism is a function of the dependencies of the surface area and transport velocity at the surface on the organism mass. For an animal characterized by a tissue density independent of mass, the surface area scales as the volume to the 2/3 power or equivalently to the 2/3 power of the organism mass. It has been argued (10) and there is empirical evidence (11) that the transport velocity for an animal scales as the 1/12 power of its mass yielding Kleiber’s law or 3/4 scaling of the metabolic rate on animal mass. This result does *not* invoke or require fractality of the underlying circulatory network (10, 12).

### Metabolic scaling of a tree

We begin by defining a few key geometrical measures of a tree as follows:

- Tree height: the height of a typical tree.
- Crown length: the difference between tree height and the height of the lowest living branch.
- Crown area: the projected area of the crown.
- Tree diameter DBH (diameter at breast height): the diameter of the stem approximately 1.3 m from the ground. We denote the tree diameter by the symbol *r*.

A power law distribution is scale-free with no characteristic scale. The height distribution of trees in a forest may have a minimum height, *h*_*min*_, which sets the unit of measure (akin to the role of a lattice parameter setting the scale for a discrete lattice in statistical mechanics). Due to resource limitations and physiological constraints (e.g. weight and gravity, wind, and structural stability) there may also be an upper cutoff *h*_*max*_. This implies that finite size corrections to a pure power law distribution need to be taken into account, resulting in the emergence of a finite, nonzero, characteristic height. Most ecologically relevant lengths in the vertical direction are proportional to this characteristic height. Here, we consider the constraint of resource availability and calculate the corrections to power law scaling and verify them with data. We find a scaling collapse of the forest data, which demonstrates the validity of the finite size scaling ansatz on determining the effects of cutoffs on the otherwise scale-free distribution through a process, which is well-acknowledged in statistical physics yet of general nature.

A tree of height *h* maximizes its metabolic rate by filling its volume, *V*, with surface elements, its leaves. Earlier work (9) has shown that the transport velocity at the surface is independent of tree mass. Thus, it follows that the metabolic rate *B* scales (the symbol ∼ denotes “scales as” and does not include constants of proportionality that would provide the right units) as the crown volume *V* (which scales as the product of the crown length and the crown area)

(1) \begin{eqnarray*} B\sim V\sim h^{1+2H}\equiv h^{D_{tree}}, \end{eqnarray*}

where *H* is the Hurst exponent (13) characterizing the scaling of the crown radius with height. The crown length is postulated to scale as the tree height accounting for the one in the exponent, and therefore, the exponent *D*_*tree*_ = 1 + 2*H*. In the simplest case of trees in tropical forests (14), *H* = 1 and *D*_*tree*_ = 3. This result arises for a tree with a maximum capacity of expanding its crown. More generally, *D*_*tree*_ seems to vary depending on the latitude and climate. Temperate forest trees expand their crown less than trees in a tropical forest and are characterized by an exponent lower than 3 (15). The exponent is larger when the resources are plentiful.

**Fig. 1.**
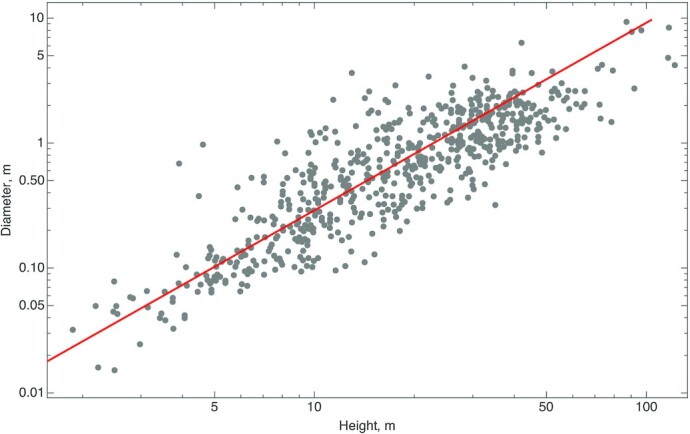
Cross plot of the diameter versus height of trees (adapted from Ref. (6)). The straight line indicates 3/2 power law scaling. Each data point represents a distinct tree species and the points plotted are record specimens or the largest recorded tree of each species.

A tree is characterized not only by its height but also by its stem diameter and empirical observations confirm that the 2 length scales are linked (6) (Fig. 1). We postulate that the allometric relationship between the metabolic rate *B* and the diameter at breast height (DBH) of a tree *r* (a quantity commonly measured for trees) is given by

(2) \begin{eqnarray*} B\sim r^\lambda . \end{eqnarray*}

Eqs. (1) and (2) provide a link between the height and the DBH of a tree. In the simplest situation, when the metabolic rate is proportional to an effective cross-sectional area, λ = 2. It is important to note that the situation is more complex because the stem comprises both active biomass (the sapwood) and nonactive biomass (the heartwood) and the composition varies with the height from the ground. The well-known structural stability analysis (6) (Fig. 1) suggests that the DBH *r* of a tree scales approximately as a power of its height, *h*, *h*^3/2^. This relation allows us to write λ as a function of the Hurst exponent *H*:

(3) \begin{eqnarray*} \lambda =\frac{2}{3}D_{tree}. \end{eqnarray*}

Note that the idealized exponents, *D*_*tree*_ = 3 and λ = 2, are consistent with Eq. (3).

We now introduce the key variable—the tree mass, *M*. Water from the ground must be transported to the leaves where the water is transpired. The mass of the tree must scale at least as the mass of the water being transported within the tree. A general theorem on transportation networks (16) states that the mass of a tree must scale at least as $h^{D_{tree}+1}$ because there are water columns of mean height *h* for each of the $h^{ D_{tree}}$ leaves. In fact, this is a lower bound for the tree mass and holds when the water is transported from the ground in a directed manner: there is no significant backtracking in the water transport route. This yields:

(4) \begin{eqnarray*} M\sim h^{D_{tree}+1}\sim r^{\lambda }h, \end{eqnarray*}

and

(5) \begin{eqnarray*} B\sim h^{D_{tree}}\sim r^{\lambda }\sim M^{D_{tree}/(D_{tree}+1)}. \end{eqnarray*}

**Fig. 2.**
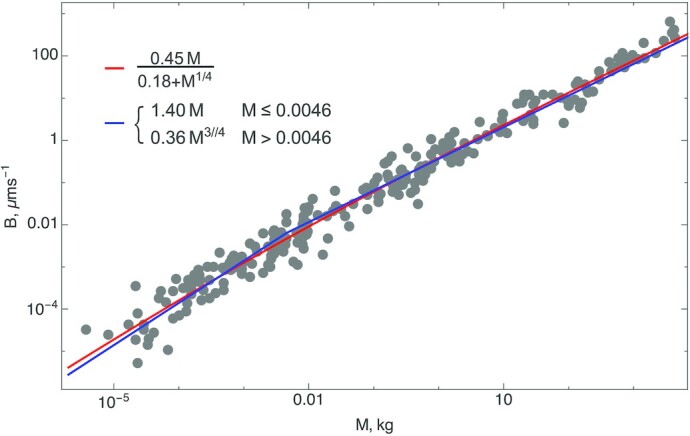
Log–log plots of metabolic rate versus mass for trees show deviations from pure power law behavior. Shigeta Mori (21) provided the tree data. At small masses the metabolic rate grows linearly with *M* and slows down to *M*^3/4^ at large masses. Both piecewise fits and crossover of exponent values are shown. Interestingly, one obtains a leading exponent indistinguishable from 3/4 if one chooses to make it an adjustable parameter (22). This figure was adapted from  (9).

When *D*_*tree*_ = 3, the metabolic rate–mass relationship is the celebrated Kleiber’s law (17) (Fig. 2). For juvenile trees, for which the entire mass is metabolically active (18–20), *B* scales as *M*, which crosses over to Kleiber’s law for large mass (9).

It is noteworthy that Kleiber’s law also holds approximately for animals, but with important underlying differences. A tree and an animal have very different geometries. A tree is approximately fractal over a range of size scales, it is rooted, its surface area or the number of leaves scales as the volume, it has no pump, and it is inhomogeneous in its mass distribution—the trunk and branches are denser than the metabolically active leaves. In contrast, an animal is not self-similar, it can move, its surface area scales in the standard Euclidean manner as the volume to the 2/3 power, it has a pump, the heart, and the mass distribution is homogeneous. In spite of their distinct geometries, animals and plants both satisfy Kleiber’s law and are metabolically equally efficient (9). Recall that the metabolic rate is proportional to product of the surface area and the velocity of heat transport at the surface. For an animal (tree), the former scales with an exponent of 2/3 (3/4) and the latter with an exponent of 1/12 (0) yielding the *same* Kleiber’s law exponent of 3/4.

The overall density of a tree is the ratio of its mass (scaling as $h^{D_{tree}+1}$) to its volume (scaling as $h^{D_{tree}}$) and scales as its height. This is a rather odd-sounding result and has simple consequences. First, it suggests that there must be a limiting height a tree can attain at which its density matches that of the trunk density. Second, the fraction of the tree volume occupied by the dense trunk and branches is bigger as the tree grows larger, demonstrating the larger overhead needed to sustain a large tree. A self-similar tree, therefore, has a natural upper cutoff scale for its fractal branching behavior.

From dimensional analysis, the characteristic time scale associated with a tree scales as *M*/*B* or equivalently is proportional to the tree height. This may be rationalized by noting that, in a stationary state, the total amount of metabolites present in the plant is proportional to the plant mass, *M*. The average time before a metabolite is used/consumed multiplied by *B* (the rate at which metabolites enter the plant) has to be equal to the total number of metabolites present. Thus, the characteristic time scale is proportional to *M*/*B* and typical rates such as the mortality rate scale as $1/h\sim 1/r^{\lambda /D_{tree}}$ in accord with empirical data (23). Finally, the cross-sectional area of a tree crown, *A*_*c*_, is predicted to scale as $A_c\sim h^{2H}=h^{D_{tree}-1}\sim r^\theta$ with θ = λ(*D*_*tree*_ − 1)/*D*_*tree*_. Plugging in the canonical values of λ = 2 and *D*_*tree*_ = 3, one predicts θ = 4/3 in accord with the results of a recent empirical and theoretical study (24).

The analysis of the scaling behavior of a single tree has built-in power laws and relationships between tree mass, tree height, trunk diameter, and the metabolic rate, so that knowledge of 1 attribute can be used to deduce the others.

### Metabolic scaling of a forest

We now turn to a forest made up of trees of different species and sizes that compete for resources (25). The NPP, or the net flux of carbon from the atmosphere into the forest per unit time, is governed by the sum of the metabolic rates of all the trees comprising the forest or, equivalently, the Leaf Area Index (LAI) or the number of leaves in the forest. Our goal is to understand the scaling of NPP with the total biomass of the forest. We invoke an optimization principle: the space available to the trees in a mature forest is fully utilized to house its leaves.

In this scenario, the total number of leaves or the metabolic rate of the forest, *B*_*forest*_, ought to be proportional to the forest volume, *Ah*_*max*_, where *A* is the total area of the forest and *h*_*max*_ is the height of the tallest tree that sets the scale of the height of the forest. For a scale-free forest, there are only 2 characteristic length scales, *h*_*min*_ and *h*_*max*_, and the relevant scale here is *h*_*max*_. This expression can also be rewritten trivially as $A h_{max}^{D_{forest}}$ with the effective forest dimension *D*_*forest*_ = 1. This is tantamount to noting that the volume of a forest of fixed area or the total number of leaves in the forest scales as the height of the forest or its tallest tree. This assumes that there is no lateral growth or that the area remains constant. The total mass of all the trees in the forest, *M*_*forest*_, would at least have an extra power proportional to *h*_*max*_, i.e. $M_{forest} \sim A h_{max}^{D_{forest}+1}$. This follows from the need for columns of water of height proportional to *h*_*max*_ supplying water in a directed manner to each of the leaves of the forest. For a forest of constant area, the metabolic rate–mass relationship becomes $B_{forest} \sim M_{forest}^{D_{forest}/(D_{forest}+1)}= M_{forest}^{1/2}$. This result corresponds to a forest of constant area behaving like an effective 1D “tree”. The scaling exponent, *D*_*forest*_, of the forest is independent of the exponent, *D*_*tree*_, characterizing the scaling of the tree but, generally, the forest dimension cannot exceed the tree dimension. The 2 are equal in the special case of a forest comprised of well-separated trees, a situation more likely to be found at high latitudes.

**Fig. 3.**
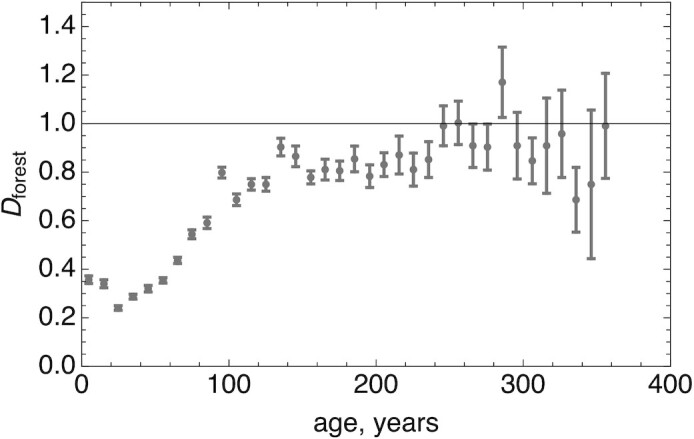
Best fit of the exponent *D*_*forest*_ characterizing the algebraic dependence of the NPP versus the biomass density as a function of forest age class for the continental United States (data taken and figure adapted from Ref. (26)). Note that these forests are managed in an even-age silviculture. As the forest matures, *D*_*forest*_ approaches the theoretically predicted value of 1 (thin horizontal line).

Figure 3 is an adaptation of the plot of the data from Kempes et al. (26) demonstrating how *D*_*forest*_ depends on the age of a forest and increases as the forest matures. One would expect that during maturation, the active mass becomes a smaller fraction of the total mass. Note that because the data of Kempes et al. refer to the scaling of the NPP on total mass and not just on the active mass, *D*_*forest*_ is typically less than or equal to 1 (Fig. 3). It is interesting to note that the forests that we will analyze have a more complex structure than those considered by Kempes et al. (26), because they comprise trees of different ages and sizes leading to better space filling.

## How Does Your Forest Grow?

We turn now to the derivation of how a forest is assembled in order to conform to the expectations in the previous section. The key assumptions of our analysis are:

- The forest is a nonequilibrium system with a continuous input of energy. For simplicity, we will assume that the forest is in a quasi-stationary state.
- The forest has trees of distinct species—we will focus on the size distribution of trees, but will not consider the differences between species. This simplification is akin to the neutrality assumption (27).
- Optimal transportation of water and metabolites governs the scaling behavior of a tree. Along the same lines, we postulate optimal resource utilization leading to maximal filling of the forest volume with leaves. Power law distributions often arise from optimal packing considerations (28, 29).

We turn now to our derivations and observations.

1. Recall that the total number of leaves in a forest is postulated to be proportional to $h_{max}^{D_{forest}}$ with the effective forest dimension *D*_*forest*_ = 1. Recall also that the number of leaves associated with a tree of height *h* scales as $h^{D_{tree}}$. Let *P*(*h*)*dh* represent the fraction of trees of the forest with height between *h* and *h* + *dh*. Thus, the total number of leaves in the forest is proportional to
  (6) \begin{eqnarray*} h_{max}^{D_{forest}} \sim \int ^{h_{max}} h^{D_{tree}} P(h)dh, \end{eqnarray*}
  yielding
  (7) \begin{eqnarray*} P(h)dh\sim h^{D_{forest}-D_{tree}-1}dh \end{eqnarray*}
  with *D*_*forest*_ = 1, leading to, upon changing variables ($M\sim h^{D_{tree}+1}$ and $r\sim h^{D_{tree}/\lambda }$),
  (8) \begin{eqnarray*} P(M)dM\sim M^{(D_{forest}-2D_{tree}-1)/(D_{tree}+1)}dM \end{eqnarray*}
  and $P(r)dr\sim r^{-(\lambda +1)+\frac{\lambda D_{forest}}{D_{tree}}}dr$.
  For a constant forest area, the characteristic height of a forest scales linearly with resource availability (14). This linear dependence does *not* hold for other otherwise equivalent tree variables such as the tree diameter or the tree mass.
2. The result above can be confirmed through an alternative derivation of *P*(*r*)*dr*, the fraction of trees with diameter between *r* and *r* + *dr*. The crown area, *A*_*c*_, of a tree scales as $h^{2H}= h^{D_{tree}-1}\sim r^\theta$, where θ = λ − λ/*D*_*tree*_. A tree of DBH *r* is associated with a distinct height *h*, which increases monotonically with *r*. The number of trees of a given size class that can fit (24) within an available constant total area scales as *r*^−θ^—note that the constancy of the area for any size class follows from an implicit assumption of *D*_*forest*_ = 1. Thus, the number of trees with size between *r* and *r* + *dr* is obtained by taking a derivative of *r*^−θ^ and is given by *P*(*r*)*dr* ∼ *r*^−(θ + 1)^*dr*. This derivation is inspired by the results of a recent study by Farrior et al. (24) of the behavior of the diameter distribution of trees comprising a forest and is in accord with our previous result, when *D*_*forest*_ = 1.
3. Our analysis bears directly on what is commonly called the energy equivalence principle (30, 31). Imagine a resource reservoir with a constant power output *B*_*res*_. Consider a set of individual trees of a given mass *M*, a height *h*, and a trunk diameter *r*. Such a reservoir can sustain an abundance of trees $N=B_{res}/B\sim M^{-D_{tree}/(D_{tree}+1)}\sim h^{-D_{tree}}\sim r^{-\lambda }$. This simple and exact observation does not tell us anything about a population of coexisting trees of differing sizes nor does it require that the energy utilized per unit time by distinct size classes is the same. This latter point has been a source of confusion in the literature. In addition, when considering diverse sizes, one has to be careful about which variable one uses to characterize the size. This could lead to distinct results because of the nonlinear scaling relationship between the different size variables.
  Eq. (7) satisfies the energy equivalence principle when *D*_*forest*_ = 1 because the product of the metabolic rate, scaling as $h^{D_{tree}}$, and the abundance of the height class, scaling as $h^{-D_{tree}}$, is independent of *h* and the height class. The key point is that this does not hold if one considers partitioning the classes along either the *r* or the *M* axes or if *D*_*forest*_ ≠ 1. There have been examples in the literature of the misleading conclusion: e.g. *P*(*r*)*dr* ∼ *r*^−λ^*dr* by noting that *B* ∼ *r*^λ^ and invoking the energy equivalence principle with the tree diameter variable (32, 33).
4. For a forest in steady state, as trees grow and graduate from one height class to the next, the mortality of trees in a given size class (governed by the mortality rate ∼1/*h*) times its abundance (scaling as $h^{D_{forest}-D_{tree}-1}$) exactly accounts for the abundance difference between neighboring classes (scaling as the derivative of the abundance or as $h^{D_{forest}-D_{tree}-2}$). This ensures that a forest in steady state remains in steady state.

Table 1 presents the predictions of our analyses for both a tree and a forest.

**Table 1.** Summary of key results. The energy equivalence principle postulating that equal energy is consumed in each tree class is satisfied only if one divides the trees into height classes and *D*_*forest*_ = 1. For simplicity, here we assume isometric scaling between active biomass and total biomass of a tree. Scaling analyses of forest structure and dynamics were first presented by West, Brown, and Enquist (32, 33).

Idealized values of exponents	*D*_*tree*_ = 3; *D*_*forest*_ = 1; λ = 2*D*_*tree*_/3
Tree height, *h*	
Scale of height of tallest tree in a forest, *h*_*max*_	
Cutoff tree diameter beyond which there are	
deviations from a power law distribution of sizes, *r*_*cut*_	
Metabolic rate, *B*	$B\sim h^{D_{tree}}\sim r^\lambda \sim M^{D_{tree}/(D_{tree}+1)}$
Tree mass, *M*	$M\sim h^{D_{tree}+1}\sim r^{\lambda (D_{tree}+1)/D_{tree}}$
Tree DBH, *r*	$r\sim h^{D_{tree}/\lambda } \sim h^{3/2}$
Crown area, *A*_*c*_	$A_c\sim h^{D_{tree}-1}\sim r^\theta$ , where θ = λ − λ/*D*_*tree*_
Structural stability	*D*_*tree*_ ∼ 3λ/2
Net primary productivity NPP, Leaf area index LAI	$\text{NPP}\sim \text{LAI}\sim h_{cut}^{D_{forest}}\sim \rho ^{D_{forest}/(D_{forest}+1)}$
Biomass density of forest (e.g. measured in kg/m^2^), *ρ*	$\rho \sim h_{cut}^{D_{forest}+1}$
Mortality rate of tree	$\sim 1/h\sim r^{-\lambda /D_{tree}}$
Growth rate of tree	*dh*/*dt* ∼ constant; $dr/dt\sim r^{1-\lambda /D_{tree}}$
Probability distribution of tree sizes in a forest	$P(h)dh\sim h^{D_{forest}-D_{tree}-1}dh$ ,
	$P(M)dM\sim M^{(D_{forest}-2D_{tree}-1)/(D_{tree}+1)}dM$ ,
	$P(r)dr\sim r^{-(\lambda +1)+\frac{\lambda D_{forest}}{D_{tree}}}dr$

## Universal Size Distribution of Tropical Forest Trees

In order to get a measure of the NPP, we need information on the size distribution of trees in a forest so we can obtain a measure of the total number of leaves in the forest. Based on just a few hypotheses, we have derived power law distributions for various tree traits. However, these are “ideal” distributions that ignore constraints such as resource limitations or the fact, mentioned before, that the tree height cannot grow indefinitely. But such distributions deviate from power laws at large sizes close to a cutoff size beyond which there are very few trees.

We carry out our analysis with the variable *r*, the trunk DBH, which is straightforward to measure empirically. Let us postulate a power law with a decay exponent, which we denote by *α*. It is straightforward to convert our expressions to any other variable including the tree height *h*. Our aim is to deduce the deviation from power law behavior for large size trees. We present 2 analytic derivations (see Supplementary Information), which yield the *same* result. Both rely on the same basic premise that there is a ceiling on the number of leaves that a forest of a given size can hold because of metabolic limitations. We find analytically that the tree diameter distribution is

(9) \begin{eqnarray*} P(r)=\frac{1}{K} r^{-\alpha }e^{-r^\lambda /r_{cut}^\lambda }, \end{eqnarray*}

where *r*_*cut*_ is a cutoff diameter governing the cross-over diameter scale beyond which the distribution deviates from a power law and there are few trees and 1/*K* is the normalization constant. The distribution (9) is obtained on postulating a Poisson tree distribution and using the results of (34) or using the maximum entropy principle  (35, 36). Moreover, the power law exponent is predicted to be α = λ + 1 − λ*D*_*forest*_/*D*_*tree*_. *α* depends on the 2 parameters *D*_*forest*_ and *D*_*tree*_, because, by Eq. (3), λ is a function of *D*_*tree*_. By considering the ideal case of *D*_*forest*_ = 1 and *H* = 1, the latter being in accord with the empirical data (see Supplementary Information), one finds that *α* = 7/3, which is close to the value derived by Farrior et al. (24) based on the empirical scaling of the crown area of a tree on its diameter (37). We, thus predict a universal diameter distribution for tropical forests with *r*_*cut*_ as the only adjustable parameter. In particular, from Eq. (9), we find that $KP(r)r^{\alpha }=e^{-r^\lambda /r_{cut}^\lambda }:=g(r/r_{cut})$, is a function of *r*/*r*_*cut*_. Thus, we predict that, with 1 adjustable parameter *r*_*cut*_ for each forest, the diameter distributions of tropical forests ought to collapse on top of each other when *g*(*x*) is plotted versus the rescaled coordinate *x* = *r*/*r*_*cut*_. We now proceed to a test of our prediction with empirical data from tropical forests around our planet.

### Comparison of theoretical predictions of diameter distribution with forest data

**Fig. 4.**
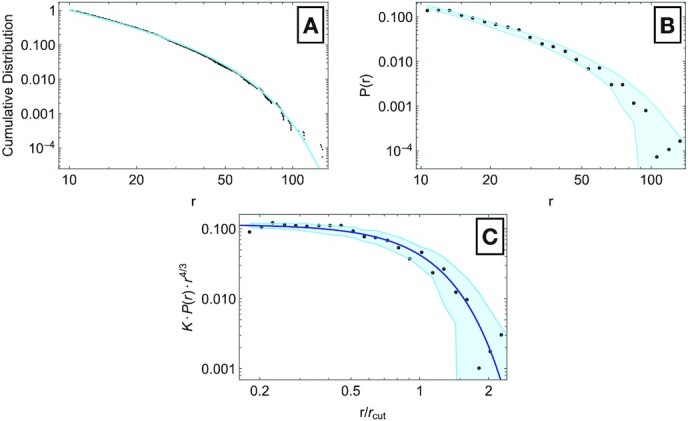
Fitting curve and scaling collapse plot for the Ranomafana forest data. (A) Empirical cumulative distribution of the tree diameters of the Ranomafana tropical forest. The light-blue curve is the fitting line obtained with *r*_*cut*_ as the only adjustable parameter. (B) We generated 100 data sets from the fitting distribution and we compared the resulting distributions with the distribution of the original forest. For each bin in the *r* coordinate, the light-blue band represents the 1-SD interval around the mean values of the 100 computer-generated data sets, whereas the black points denote the empirical distribution *P*(*r*) of the Ranomafana forest. (C) The black points have been obtained via the rescaling of the empirical diameter distribution of the tropical forest, whereas the light-blue curve represents the 1-SD interval around mean values within each logarithmic bin of the rescaled *r*/*r*_*cut*_ coordinates. The blue curve is our prediction for the scaling collapse curve, $e^{-x^\lambda }$. Note that the collapse exponent is 4/3 because the rescaled coordinates are presented in a logarithmic scale. Were one to use a linear scale for the *x*-axis, we would need to instead compute *K* · *P*(*r*) · *r*^7/3^.

In order to test our predictions, we analyzed data from 14 tropical forests provided by the Tropical Ecology, Assessment and Monitoring (“TEAM”) Network (http://www.teamnetwork.org/, accessed in May 2021). For each forest, we considered all trees with diameters (DBH) greater than 10 cm and smaller than 500 cm. By fitting the theoretical distribution (9) to the data, we found the set of 14 *r*_*cut*_ values, which best describe the empirical forest diameter distributions (see Supporting Information). In Fig. 4, we show the results for the Ranomafana forest in Madagascar. Panel A shows the best-fit distribution (light-blue) curve against the forest data (black dots). Remarkably, the distribution (9) with just 1 adjustable parameter captures the behavior of the empirical tree diameter distribution. As an additional check, we generated 100 data sets from the fitted distribution and we compared the original and the computer-generated diameter distributions (Panel B of Fig. 4). We then binned both the original and computer-generated data along the DBH axis and computed the average and SD among the 100 computer data sets at each bin. The black points in the figure are empirical values of *P*(*r*) of the Ranomafana forest, whereas the light-blue band corresponds to the 1-SD interval around the mean values of *P*(*r*) for the generated data (see Supplementary Information for the graphics of the other 13 forests we analyzed). Finally, Panel C of Fig. 4 shows the plot of the rescaled distribution *KP*(*r*)*r*^α^ ≔ *g*(*r*/*r*_*cut*_) against the scaled coordinates *r*/*r*_*cut*_. We find that both the original points (black dots) and the generated data (light-blue band) are in agreement with the predicted curve $e^{-x^\lambda }$ (blue curve).

**Fig. 5.**
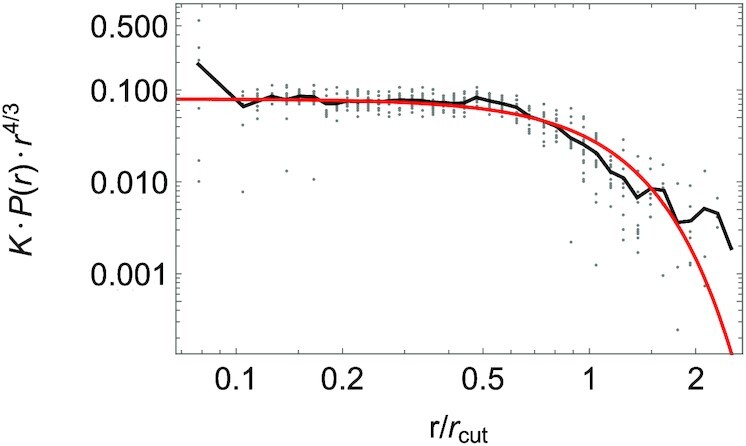
Scaling collapse of the empirical diameter distributions of the 14 tropical forests. The gray dots are empirical rescaled points (*r*/*r*_*cut*_, *K* · *P*(*r*) · *r*^4/3^) for each forest, where *r*_*cut*_ is the best-fit value of the single adjustable parameter for each forest, *K* is the inverse of the normalization constant of the corresponding *P*(*r*) distribution and the exponent 4/3 = α − 1 due to the choice of logarithmic binning). The black curve depicts the mean values of empirical points within each bin of the rescaled *r*/*r*_*cut*_ coordinates, while the red curve represents our prediction for the scaling collapse curve, $e^{-x^\lambda }$.

In Fig. 5, we show the scaling collapse of the empirical tree diameter distributions of all the tropical forests. Interestingly, the theoretical curve $e^{-x^\lambda }$ (the red line in Fig. 5) accurately captures the qualitative trends of the data. The black curve in Fig. 5 represents the average value among the 14 forest data points, depicted as gray dots, along the binned *r*/*r*_*cut*_ axis. The key lesson is that the tail of the diameter distribution contains valuable information on the dependence of the metabolic energy of a tree on its diameter. However, the statistics in the tail of the distribution is poor. Interestingly, the scaling analysis links the power law exponent to the deviation of the distribution from power law behavior for large diameters.

## Scaling of the NPP with Total Biomass Density *ρ* of a Forest

We are now ready to study the metabolic scaling of the forest and its dependence on the dimensionalities of a forest and the trees comprising it. We switch to the height variable from the DBH variable, *r*, to obtain the analog of Eq. (9). $\tilde{P}$ is a function different from the function *P* of the DBH variable. The specific form of the distribution function arises because the characteristic energy of a tree of height *h*_*cut*_ scales as $h_{cut}^{D_{tree}}$. The NPP and the total biomass density, *ρ*, scale as averages of a tree’s number of leaves, $h^{D_{tree}}$, and a tree’s mass, $h^{D_{tree}+1}$, respectively, over all the trees in the forest:

(10) \begin{eqnarray*} \text{NPP}\sim \int _{h_{min}}^{h_{max}}h^{D_{tree}}\tilde{P}(h)e^{-\left(\frac{h}{h_{cut}}\right)^{D_{tree}}}dh \sim h_{cut}^{D_{forest}}, \end{eqnarray*}

(11) \begin{eqnarray*} \rho \sim \int _{h_{min}}^{h_{max}}h^{D_{tree}+1}\tilde{P}(h)e^{-\left(\frac{h}{h_{cut}}\right)^{D_{tree}}}dh \sim h_{cut}^{D_{forest}+1}, \end{eqnarray*}

where *h*_*min*_ and *h*_*max*_ are the heights of the smallest and largest tree classes, respectively (we assume that *h*_*min*_ ≪ *h*_*max*_). In typical cases, one would expect that *h*_*cut*_ is less than or of order *h*_*max*_ and either of these heights represent the characteristic tree height in the forest. For *D*_*forest*_ < *D*_*tree*_, $\tilde{P}(h)\sim h^{D_{forest}-D_{tree}-1}$ yielding $\text{NPP}\sim h_{cut}^{D_{forest}}$, $\rho \sim h_{cut}^{D_{forest}+1}$, and therefore, $\text{NPP}\sim \rho ^{D_{forest}/(D_{forest}+1)}$. When *D*_*forest*_ = *D*_*tree*_, any $\tilde{P}(h)\sim h_{cut}^{\mu -1}h^{-\mu }$ with *μ* < 1 is satisfactory and when *D*_*forest*_ > *D*_*tree*_, none is.

It is intriguing that the *B* − *M* scaling of a tree and NPP − *ρ* scaling of a forest have similar functional forms with the exponent of the forest being at most as effective as a single tree. It is especially noteworthy that the NPP of a forest is primarily controlled by the single length scale *h*_*cut*_. Kempes et al. (26) have also proposed a theory that obtains a scaling relationship between NPP and the biomass density of a forest. Our analysis is distinct from their work.

## Conclusion

Forests are complex many-body systems spanning multiple scales. We demonstrate how general trends and patterns emerge in forests from scale-free behaviors of their various components. By enforcing geometrical and physical constraints, our analysis of large bodies of empirical data on metabolic scaling of trees and forests suggest a notable underlying simplicity despite the high degree of structural and functional diversity of these key ecosystems. We analytically derive a universal tree size distribution for tropical forests. Our theory captures the scaling behavior of empirical data from 14 tropical forests.

Our work has implications for understanding carbon sequestration and in climate science. The quasi-equilibrium state of a community depends sensitively on the value of *D*_*forest*_. When it is close to 1, the net-primary productivity reaches a limiting value with a maximal number of leaves and biomass in relation to the available resources. The amount of carbon stoked within the community attains its maximum value in this limit. Furthermore, in this situation, the forest community reaches a quasi-equilibrium state, with a negligible net flux of carbon dioxide (forest uptake-emissions). In contrast, when *D*_*forest*_ < 1, the forest behaves as a sink of carbon dioxide with an overall net community growth (measured by the increase in the total biomass) with time. The evapotranspiration of the forest is large as is the dissipation of latent heat. The forest acts as a cooling system for the atmosphere with a large water cycle characterized by water uptake from the soil followed by evaporation and condensation. Such information is vital for understanding the role of forests in mitigating the carbon dioxide increase in the atmosphere and in determining the effects of deforestation causing additional atmosphere warming.

## Supplementary Material

pgac008_Supplemental_Files

## Data Availability

The tree data presented in Fig. 2 were kindly provided by Shigeta Mori. The data presented in Fig. 3 was adapted from a paper on arXiv by Kempes et al. (https://arxiv.org/abs/1506.01691), which also provided inspiration for this work. The tropical forest data were provided by the Tropical Ecology Assessment and Monitoring (TEAM) Network.
