# Supplementary material for: Seeing the forest for the trees through metabolic scaling
Source: PNAS Nexus. 2022 Mar 10;1(1):pgac008. doi: 10.1093/pnasnexus/pgac008 (PMC9802057; doi:10.1093/pnasnexus/pgac008)
Supplement: pgac008_Supplemental_Files [file pgac008_supplemental_files.zip › PNASNEXUS-PNASNEXUS-2021-00158-s01.pdf]

Supplementary Information for

Seeing the forest for the trees through metabolic scaling

Igor Volkov, Anna Tovo, Tommaso Anfodillo, Andrea Rinaldo, Amos Maritan*, Jayanth R. Banavar*

* amos.maritan@unipd.it, banavar@uoregon.edu

Analytic derivations for deviations of size distributions of forest trees from power law behavior

Our aim is on deducing the deviation from power law behavior for large size trees. We begin by presenting two analytic derivations, which yield the *same* result and relying on the same basic premise that there is a ceiling on the number of leaves that a forest of a given size can hold.

Derivation analogous to standard statistical physics

It has been suggested that complex dynamical ecosystems may self-organize [1] and their behavior might mimic the behavior of a physical system near a phase transition [2]. Here we will apply the analysis in Ref. [2] to the derivation of the size distribution of trees.

Let us partition the trees into size bins of equal width δ (the kth bin is centered around $r_k = \delta k$). The number of trees in each bin is expected to follow Poisson statistics [3] with an average proportional to $r_k^{-\alpha}$:

$$P_k(n) \sim \frac{r_k^{-\alpha n}}{n!}. \tag{1}$$

The above distribution may be rationalized by assuming that the probability p of a tree to appear in a given bin is proportional to the abundance of that bin. The probability of n trees in the bin is proportional to p^n. The factor $1/n!$ follows from noting that the trees are distinguishable and they can be in $n!$ possible arrangements within the bin.

In analogy with physics, a tree can be ascribed with an energy which is proportional to the number of its leaves, $\varepsilon_k = r_k^{\lambda}$. Now let us introduce a "ceiling" on the number of leaves the forest can hold, E_{max}. This ceiling can be expressed as

$$E_{max} = T + \langle E \rangle, \tag{2}$$

where $\langle E \rangle$ is the number of leaves in the forest and T is the remaining leaf capacity. The presence of the ceiling leads to the following joint size distribution of trees:

$$P(n_1, n_2, \dots) = \frac{1}{Q} \prod_k P_k(n_k) \Theta\left(E_{max} - \sum_i \varepsilon_i n_i\right), \tag{3}$$

where $\Theta(x)$ is a Heaviside theta function (zero for $x < 0$ and 1 otherwise) and Q is a normalization factor (also called a partition function in statistical physics [4]):

$$\begin{aligned} Q = \sum_{\{n_k\}} \prod_k P_k(n_k) \Theta\left(E_{max} - \sum_i \varepsilon_i n_i\right) = \\ \sum_{\{n_k\}} \prod_k P_k(n_k) \int_\gamma \frac{dz}{2\pi i z} e^{z(E_{max} - \sum_{j=1}^{S} \varepsilon_j n_j)} = \\ \int_\gamma \frac{dz}{2\pi i z} e^{zE_{max} - \sum_k h(z\varepsilon_k)}, \end{aligned} \tag{4}$$

where $e^{-h(\beta)} = \sum_n e^{-n\beta} P(n)$ and the contour γ is parallel to the imaginary axis with all its points having a fixed real part z_0 (i.e. $z \in \gamma \Leftrightarrow z = z_0 + iy, -\infty < y < +\infty$). The integral is independent of z_0 provided z_0 is positive [5].

We evaluate the integral in Eq (4) by the saddle point method [5] by choosing z_0 in such a way that the maximum of the integrand of Eq (4) occurs when $y = 0$:

$$E_{max} - \frac{1}{z_0} = \sum_k \varepsilon_k h'(z_0 \varepsilon_k), \tag{5}$$

where the prime indicates a first derivative with respect to the argument. Note that the rhs is simply $\sum_k \varepsilon_k \langle n_k \rangle$ with the average taken with the weight $P_k(n_k) \exp[-z_0 \varepsilon_k n_k]$.

Comparing Eq (5) with Eq (2), one can make the identification $z_0 = 1/T$ and therefore

$$Q \sim \sum_{\{n_k\}} \prod_k P(n_k) \exp(-\varepsilon_k n_k / T). \tag{6}$$

Note that the leaf imbalance T controls the relative abundance of the trees in the bins, i.e. plays a role analogous to the temperature of a physical system.

As a result, the joint distribution becomes

$$P(n_1, n_2, \dots) \sim \prod_k P_k(n_k) e^{-\varepsilon_k n_k / T} \sim \prod_k \frac{(r_k^{-\alpha} e^{-\varepsilon_k / T})^{n_k}}{n_k!} \tag{7}$$

and the average number of trees in the kth bin abundances is given by

$$\langle n_k \rangle \sim r_k^{-\alpha} e^{-\varepsilon_k / T} = r_k^{-\alpha} e^{-r_k^\lambda / r_{cut}^\lambda}. \tag{8}$$

Here we introduced a characteristic diameter $r_{cut} = T^{1/\lambda}$ above which the effects of the presence of the ceiling become non-negligible.

Derivation based on the principle of maximum entropy

We turn now to an independent derivation of the deviation of the tree diameter distribution from a pure power law, which yields the same result as before. This derivation is based on the principle of maximum entropy [6, 7].

Let $P(r)$ denote the probability distribution of tree diameters r. Without imposing the total leaf capacity of the forest, this probability is taken to be a power law: $P_0(r) = K r^{-\alpha}$ for $r > r_{min}$ where $K = (\alpha - 1) r_{min}^{\alpha - 1}$ is the normalization constant.

The Kullback-Leibler entropy [8] is defined as

$$D_{KL} = \int P(x) \ln \frac{P(x)}{P_0(x)} dx \tag{9}$$

where P_0 is the *a priori* probability. Indeed, the minimization of D_{KL} with respect to P with only the normalization constraint

$$\int P(x) dx = 1 \tag{10}$$

implies that $P = P_0$. In the presence of the leaf capacity, we have an additional constraint, which is proportional to

$$N \int P(x) x^\lambda dx \tag{11}$$

(here N is the total number of plants in the forest). The minimization of (9) with the constraints (10) and (11) leads to

$$P(r) \sim r^{-\alpha} e^{-\beta r^\lambda} \tag{12}$$

which is the same as that derived earlier with $\beta = 1/r_{cut}^\lambda$.

Testing the hypothesis $H = 1$ against empirical data

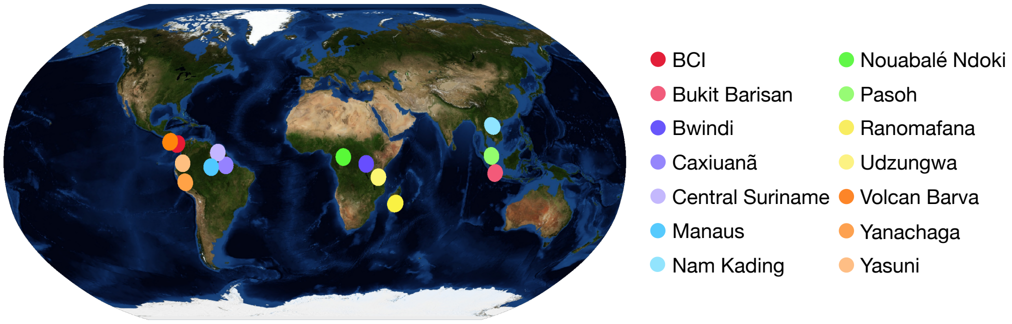

Figure S1. Map showing the locations of the fourteen tropical forests used in our study. The data were kindly provided by the Tropical Ecology, Assessment and Monitoring ("TEAM") Network (http://www.teamnetwork.org/ accessed in May 2021).

In order to test whether the choice of $H = 1$ is sensible for our tropical forest data (see Figure S1), we constructed an iterative algorithm which works as follows. Let us call $P(r|H, r_{cut})$ the empirical tree diameter distribution, where we highlighted the dependence on the two independent parameters H and r_{cut}. Indeed, if we set the canonical value $D_{forest} = 1$ and recall Eq (3) of the main text expressing $\lambda = \lambda(H)$ as a function of H, we find that $P(r|H, r_{cut})$ has the form

$$P(r|H, r_{cut}) = \frac{1}{K} r^{-\alpha(H)} e^{-(r/r_{cut})^{\lambda(H)}}, \tag{13}$$

where $1/K$ is the normalization constant and the power-law exponent α is a function of H:

$$\alpha(H) = \lambda(H) + 1 - \frac{\lambda(H)}{1 + 2H}. \tag{14}$$

Each iteration i of our algorithm consists of two steps:

- Step 1: given a value H_{i-1} of the Hurst exponent, we fit $P(r|H_{i-1}, r_{cut})$ against the empirical tree diameter distribution with only r_{cut} as adjustable parameter.
- Step 2: given the best-fitting value $r_{cut,i}$ found at step 1, we fit $P(r|H, r_{cut,i})$ against the empirical tree diameter distribution, this time with only H as adjustable parameter. We call H_i the corresponding best-fitting value.

Then, by setting an initial value H_0, after n iterations the algorithm returns two sequences $r_{cut,1}, \ldots, r_{cut,n}$ and $H_1, \ldots, H_n$. We applied the algorithm to each of the 14 tropical forests by setting $H_0 = 0.85$ and after 50 iterations we obtained the two sequences shown in Figure S2. Remarkably, while the $r_{cut,i}$ sequences converge to different values of the cutoff parameter which thus results to be forest-dependent, all H_i sequences converge to 1, the canonical value of the Hurst exponent for tropical forests. This result justifies our choice of $H = 1$ when performing the collapse analysis described in the main text.

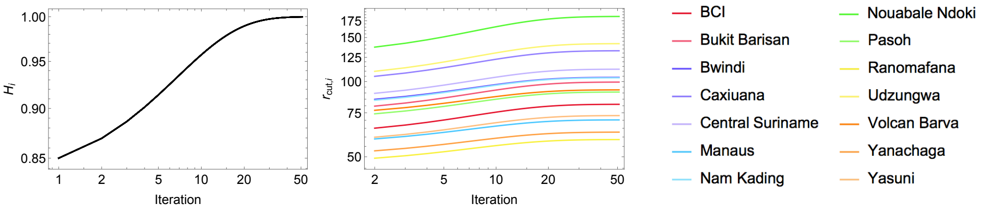

Figure S2. Hurst exponent and cutoff parameter sequences obtained via the recursive algorithm on the fourteen tropical forests. While the $r_{cut,i}$ sequences converges to specific values for different data sets (right panel), all H_i sequences smoothly converge to 1, the canonical value for tropical forests (left panel).

In Table S1, we display the values of the fourteen r_{cut} parameters obtained via maximum likelihood by fitting our theoretical distribution $P(r|1, r_{cut})$ to the empirical tree diameter data of the tropical forests. Finally, Figure S3 shows how these parameters allow us to capture the behaviour of the empirical diameter distributions of the remaining thirteen tropical forests. The fitting curve and scaling collapse plot for the Ranomafana forest data is presented in the main text.

Table S1. Best-fit r_{cut} parameters of the fourteen tropical forests obtained by fitting data to theory via maximum likelihood estimation.

**Forest**	$\mathbf{r_{cut}}$ **(cm)**
BCI	81.1
Bukit Barisan	99.5
Bwindi	104.0
Caxiuana	132.8
Central Suriname	112.1
Manaus	70.2
Nam Kading	103.5
Nouabalé Ndoki	182.2
Pasoh	90.8
Ranomafana	58.6
Udzungwa	141.8
Volcan Barva	92.6
Yanachaga	62.8
Yasuni	73.1

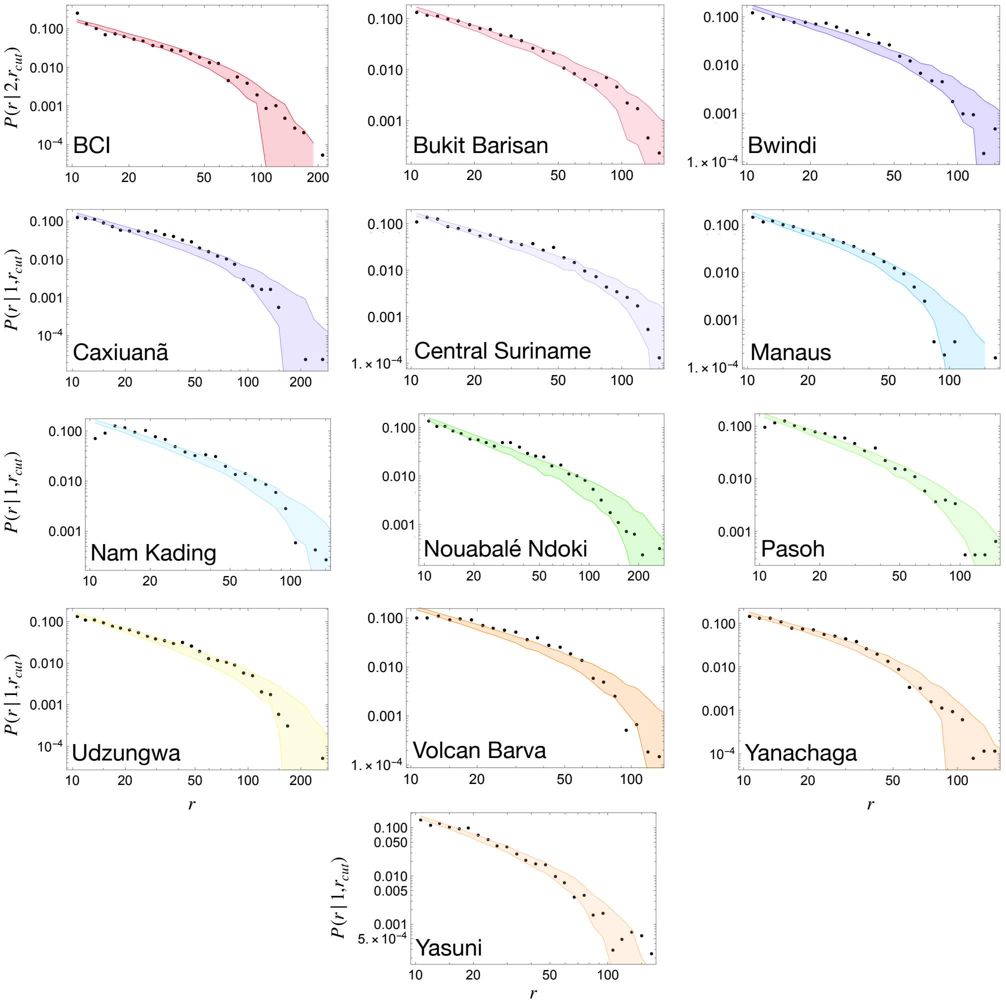

Figure S3. Fits of forest data with $H = 1$. For each forest, we generated 100 data sets from the distributions $P(r|1, r_{cut})$, where r_{cut} are the best-fit parameters for each forest obtained via maximum likelihood on forest diameter data (see Table S1). The coloured bands represent the one-standard deviation intervals around the mean values of hundred sets of computer generated data, which we compare with the distribution of the original data of each forest (black points). The corresponding plot for the Ranomafana forest is presented in the main text.

References

1. Solé R, Bascompte J. Self-Organization in Complex Ecosystems.(MPB-42). vol. 42. Princeton University Press; 2012.

2. Volkov I, Banavar JR, Maritan A. Organization of ecosystems in the vicinity of a novel phase transition. Physical review letters. 2004;92(21):218703.

3. Rao CR. In: Patil GP, Pielou EC, Waters WE, editors. Statistical Ecology Vol. 1: Spatial Patterns and Statistical Distributions. University Park, PA: The Penn. State Univ. Press; 1971. p. 131–142.

4. Feynman RP. Statistical Mechanics. Reading, MA: Addison-Wesley; 1998.

5. Morse PM, Feshbach H. Methods of Theoretical Physics, Part I. New York, NY: McGraw-Hill Book Company, Inc.; 1953.

6. Volkov I, Banavar JR, Maritan A. A novel ensemble in statistical physics. Journal of statistical physics. 2006;123(1):167–180.

7. Harte J, Newman EA. Maximum information entropy: a foundation for ecological theory. Trends in ecology & evolution. 2014;29(7):384–389.

8. Kullback S, Leibler RA. On information and sufficiency. The annals of mathematical statistics. 1951; p. 79–86.
